# Top-down control of vestibular inputs by the dorsolateral prefrontal cortex

**DOI:** 10.1007/s00221-023-06722-6

**Published:** 2023-10-30

**Authors:** Brendan McCarthy, Sudipta Datta, Gianni Sesa-Ashton, Rebecca Wong, Luke A. Henderson, Tye Dawood, Vaughan G. Macefield

**Affiliations:** 1 Baker Heart and Diabetes Institute, Melbourne, VIC Australia; 2 Baker Department of Cardiometabolic Health, The University of Melbourne, Melbourne, VIC Australia; 3 School of Medical Sciences (Neuroscience), Brain and Mind Centre, The University of Sydney, Sydney, NSW Australia; 4 Department of Neuroscience, Central Clinical School, Monash University, 99 Commercial Road, Melbourne, VIC 3004 Australia

**Keywords:** Dorsolateral prefrontal cortex, Insula, Nausea, Transcranial electrical stimulation, Vestibular system

## Abstract

The vestibular apparatus provides spatial information on the position of the head in space and with respect to gravity. Low-frequency sinusoidal galvanic vestibular stimulation (sGVS), a means of selectively changing the firing of vestibular afferents, induces a frequency-dependent perception of sway and, in some individuals, induces nausea. Given that vestibular afferents project to the insular cortex—which forms part of the vestibular cortex—and that the insula receives inputs from the dorsolateral prefrontal cortex (dlPFC), we tested the hypothesis that electrical stimulation of the dlPFC can modulate vestibular inputs. Sinusoidal electrical stimulation (± 2 mA, 0.08 Hz, 100 cycles) was delivered via surface electrodes over (1) the mastoid processes alone (sGVS), (2) electroencephalogram (EEG) site F4 (right dlPFC) and the nasion or (3) to each site concurrently (sGVS + dlPFC) in 23 participants. The same stimulation protocol was used in a separate study to investigate EEG site F3 (left dlPFC) instead of F4 in 13 participants. During sGVS, all participants reported perceptions of sway and 13 participants also reported nausea, neither sensation of which occurred as a result of dlPFC stimulation. Interestingly, when sGVS and dlPFC stimulations were delivered concurrently, vestibular perceptions and sensations of nausea were almost completely abolished. We conclude that the dlPFC provides top-down control of vestibular inputs and further suggests that dlPFC stimulation may provide a novel means of controlling nausea.

## Introduction

The vestibular apparatus provides spatial information on the position of the head in space and with respect to gravity. Low-frequency sinusoidal galvanic vestibular stimulation (sGVS) is a means of selectively altering vestibular afferent firing patterns without moving one’s head and induces a frequency-dependent perception of cyclic lateral sway. This illusory perception is often described as ‘swaying in a hammock’ or ‘rocking in a boat’, and is not associated with actual movement or changes in the activity of leg muscles in seated or passively standing participants (Bent et al. 2007, 2013; Knellwolf et al. 2016). As a result of this, sGVS is capable of inducing nausea in some individuals.

Various means of vestibular stimulation including galvanic (both with a sinusoidal current as well as a direct one) have been employed concurrently to functional magnetic resonance imaging (fMRI) to identify key structures of the vestibular system (zu Eulenburg et al. 2012). One such key structure is known as the parieto-insular vestibular cortex (PIVC). Whilst readily identifiable in non-human primates (Akbarian et al. 1988; Chen et al. 2010; Guldin and Grüsser 1998), determining the human equivalent has produced varying results (Lobel et al. 1998, 1999; Fasold et al. 2002; McCarthy et al. 2023). Nevertheless, it is generally accepted that the core human vestibular cortex comprises the posterior insula (and, more specifically, the granular insula), retroinsular and parietal operculum (Ibitoye et al. 2023; zu Eulenburg et al. 2012). Neurones within the primate PIVC respond to vestibular and proprioceptive stimulation (Guldin and Grüsser 1998) and investigations in macaque and squirrel monkeys present the PIVC as a key integration site within the wider vestibular system for spatial, proprioceptive and optokinetic information (Akbarian et al. 1993; Chen et al. 2010; Guldin and Grüsser 1998). Furthermore, in rhesus monkeys, the PIVC is active during translational and rotational movements of the head or body (Chen et al. 2010).

The insular cortex has extensive connexions to the prefrontal, temporal and parietal cortices, as well as components of the limbic system. Additionally, it forms part of the interoceptive cortex—monitoring the internal state of the body and integrating this with information from the external environment (Sliz and Hayley 2012). The insula has widespread connexions with other association cortices (Mesulam and Mufson 1982), including the dorsolateral prefrontal cortex (dlPFC), albeit through the anterior part of the insula (Cieslik et al. 2013; Jung et al. 2022). The dlPFC is a complex area of the frontal lobe primarily involved in higher-order, multi-sensory processing and executive control, and is involved in cognitive and emotional processing (Brunoni and Vanderhasselt 2014; Golkar et al. 2012; Krummenacher et al. 2010; Seminowicz and Moayedi 2017; Staudinger et al. 2011). Importantly, fMRI studies have shown that activation of vestibular afferents through sGVS causes signal changes in the posterior insula and temporoparietal junction, but also activation in Brodmann’s areas 44, 6 and 9, the latter of which forms the main part of the dlPFC (Lobel et al. 1998, 1999).

Results such as these may be somewhat unexpected, but it has been well established that the dlPFC can regulate behaviour and modulate other structures through inhibitory control. For example, impulse control in economic exchange games is associated with left dlPFC activity (Steinbeis et al. 2012) and, in psychiatric disorders, such as major depressive disorder, dlPFC dysfunction impairs inhibitory control (Hamilton et al. 2013). Besides regulating behaviour, the dlPFC is known to regulate incoming sensory information, such as noxious information (Youssef et al. 2016). Given this, it is likely that the dlPFC may regulate other sensory modalities including vestibular information. Indeed, transcranial magnetic stimulation of the dlPFC has been reported to reduce fMRI activations in Brodmann area 13 in humans, corresponding to the posterior insula (Ye et al. 2022). Individuals with conditions involving disruption of the vestibular system, such as persistent postural–perceptual dizziness, display reduced dlPFC volumes (Wurthmann et al. 2017), potentially underpinning reduced inhibitory output onto vestibular-processing brain regions including the insula.

Given this, we sought to evaluate the effects of dlPFC stimulation on vestibular illusions produced by sGVS. To modulate dlPFC function, we used transcranial alternating current stimulation (tACS), which alters membrane excitability but is not believed to directly generate action potentials (Colzato et al. 2017; Knotkova et al. 2019). This was administered at the same low-frequency stimulation (± 2 mA, 0.08 Hz, 100 cycles) as was used to stimulate vestibular afferents during sGVS, delivering the stimuli separately and together in phase. We hypothesised that tACS of the dlPFC would inhibit the perception of swaying evoked by sGVS.

## Methods

Studies investigating the right dlPFC at electroencephalogram (EEG) site F4 were performed on 23 young adults, hereafter named ‘right dlPFC participants’ [15 males and eight females; age: 21–32 years, mean ± standard deviation (SD), 23.3 ± 2.8 years; height: 155–193 cm, 171.6 ± 8.8 cm; weight: 53–117 kg, 71.6 ± 13.7 kg]. This study was approved by the Human Research Ethics Committee (HREC) of Western Sydney University (HREC approval H11010). Studies investigating the left dlPFC at EEG site F3 were performed on 13 young adults, hereafter named ‘left dlPFC participants’ (seven males and six females; age: 21–39 years, 26.4 ± 4.3 years; height: 155–185 cm, 167.5 ± 9.2 cm; weight: 40–96 kg, 65.2 ± 15.5 kg). This study was approved by the Human Research Ethics Committee (HREC) of the Alfred Hospital (HREC approval 62155). Both investigations were endorsed by Governance of the Baker Heart and Diabetes Institute and conformed to the Declaration of Helsinki, with all participants providing informed written consent.

### Stimulation procedures

Participants lay semi-recumbent in a chair with their backs at 45° and their legs supported horizontally. To locate the region of the dlPFC, an EEG cap was placed over the head and the standard 10–20 EEG position F3 or F4, corresponding to the left or right dlPFC respectively, was marked. After removing the cap, 35 mm adhesive hydrogel Ag–AgCl electrodes (Covidien, Ireland) were placed over the dlPFC site as well as over the nasion, which served as the reference. The same type of electrode was placed over left and right mastoid processes for use in stimulation of the vestibular apparatus. Additional conductive cream was placed under the electrodes to optimise contact, whilst an elastic headband or gauze wrapped around the participant’s head ensured the electrodes remained in place.

Following a four-minute baseline period, sinusoidally modulated stimuli (± 2 mA, 0.08 Hz) were delivered to the dlPFC alone, mastoid processes alone, or both the dlPFC and mastoid processes concurrently via an isolated constant current stimulator (Linear Stimulus Isolator, World Precision Instruments, FL, USA) connected to the Ag–AgCl electrodes placed on the corresponding areas and controlled by PowerLab (ADInstruments, Sydney, Australia). Participants received: (1) sGVS via Ag–AgCl electrodes over the mastoid processes (anode on the right), (2) tACS of the dlPFC (with the anode on the nasion), and (3) combined stimulation. Stimulus trains were delivered in a random order and a four-minute rest period separated each of the stimulation protocols. Each period of stimulation was delivered for 100 cycles, and at 0.08 Hz each stimulus set lasted for ~ 21 min. During the entire stimulation protocol, participants tracked any perceived movements using a linear potentiometer (Response Meter, ADInstruments, Sydney, Australia), with which they practised moving before the stimulation began. Participants were instructed to move the potentiometer to the central position immediately before the stimulus began and to track any perceived motion by sliding the potentiometer to the right or left—a perceived rightwards or leftwards sway would be tacked with rightwards or leftwards movement, respectively, on the potentiometer. The sine wave corresponding to the stimulus was recorded alongside the potentiometer signal. Participants were also asked to describe any sensations, including nausea, in real-time. Nausea was rated on a 10-point sliding scale from 0 (no nausea) to 10 (maximal nausea; feels as if they will vomit).

### Data analysis

All statistical analyses were performed using Prism 9.1.2 for Macintosh (GraphPad Software, USA). Data were tested for normality using Kolmogorov–Smirnov Normality tests. Kruskal–Wallis tests with a Dunn’s multiple comparisons test was performed on the perceived motion data of the right dlPFC experiments. Friedman tests with a Dunn’s multiple comparisons test was performed on the perceived motion data of the left dlPFC experiments. The same tests were conducted on the perceived nausea data of the participants who experienced nausea in order to determine whether or not significant differences were found between stimulation protocols. Data are presented as mean ± SD.

## Results

**Fig. 1:**
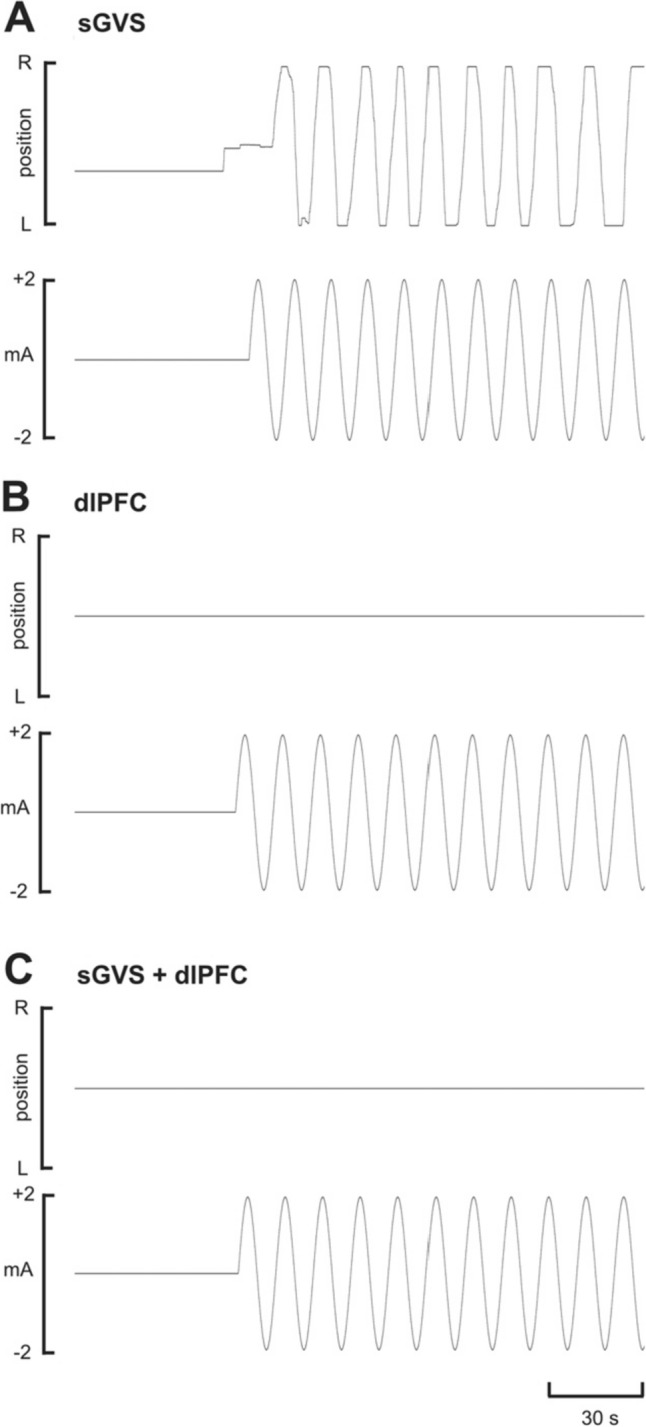
Perceptions of side-to-side motion (potentiometer signal in the top traces of each panel) during vestibular stimulation alone (sGVS), stimulation of the right dlPFC alone (dlPFC) or combined stimulation (sGVS + dlPFC) in one participant. The sine wave in the lower traces of each panel represents the actual stimulus delivered to each site

Binaural sGVS induced a robust sensation of sway in all 20 participants who underwent the stimulation protocol during stimulation of the right dlPFC (in three cases technical issues prevented sGVS from being administered alongside the other two stimulation protocols, reducing the cohort from 23 to 20). All 13 participants who underwent sGVS during the left dlPFC experiments likewise reported a perception of sway. These perceptions were indicated by participant-reported feedback using a linear potentiometer, a sample recording of which is shown in Fig. 1A. The participant tracked the perceived motion to the right or the left which corresponded to positive and negative phases of the applied stimulus, respectively. Of the 20 right dlPFC participants, it is interesting to note that five described the illusions as more leftward-dominant, whereas none indicated a predominantly rightwards sway. Similarly, in the 13 left dlPFC participants, three reported a swaying sensation that was more leftward-dominant and none indicated the same for the right. In 11 of the 20 right dlPFC experiments and nine of the 13 left dlPFC experiments, participants reported dissipation of the vestibular illusions over time, though this can be attributed to habituation to the stimulus over the ~ 21-min stimulation protocol.

Conversely, tACS of the right and left dlPFC did not produce any illusory movements in any of the 22 (technical difficulties prevented dlPFC stimulation during one of the right dlPFC experiments) or 13 participants, confirmed by the absence of signalling of perceived motion via the linear potentiometer (Fig. 1B). Importantly, when dlPFC stimulation was applied at the same time and in the same phase as binaural sGVS, perceptions of sway which occur with sGVS alone were abolished in all 23 right dlPFC participants from the onset (Fig. 1C). The lack of reporting could not be attributed to habituation or distraction. A similar, but not identical, result was found with combined stimulation of the vestibular apparatus and left dlPFC. Four of the 13 participants reported a form of sway, but in each case, the participant described it upon the conclusion of the experiment as distinct and greatly diminished in comparison to the sway they perceived during sGVS. It should be noted that for all stimulus conditions, participants reported an initial burning or tingling sensation under the stimulating electrodes. These sensations were generally well tolerated and would usually abate within approximately two minutes of each set of the ~ 21-min stimulation period.

**Fig. 2:**
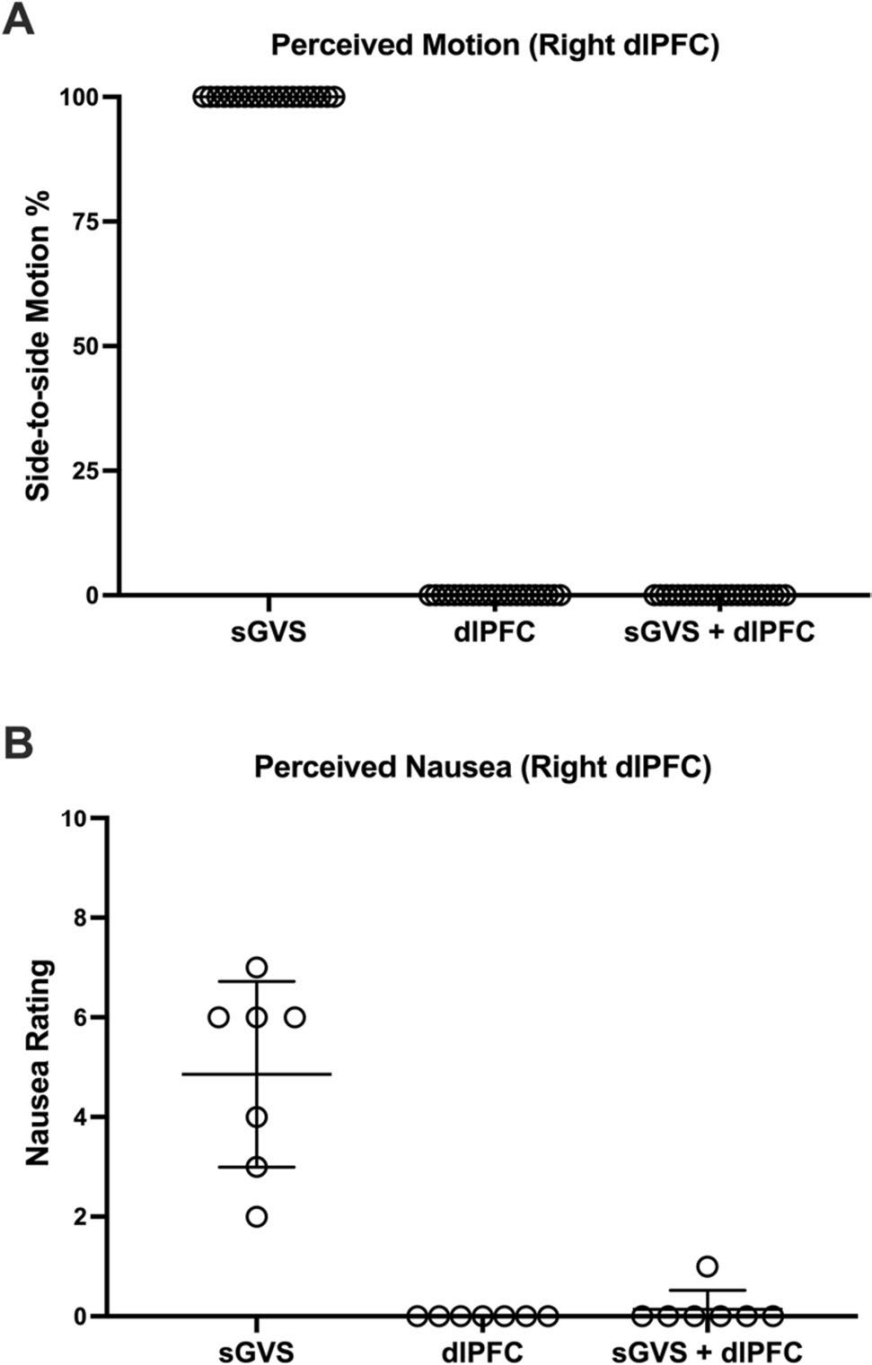
Perceptions of side-to-side motion (**A**) and nausea (**B**) during vestibular stimulation alone (sGVS) (*n* = 20), stimulation of the right dlPFC alone (dlPFC) (*n* = 22) or combined stimulation (sGVS + dlPFC) (*n* = 23). The prevalence of perceived motion was significantly different between sGVS and dlPFC stimulation (*P* < 0.0001) as well as between sGVS and sGVS + dlPFC stimulation (*P* < 0.0001). For the nausea ratings, mean ± SD from the seven participants who experienced nausea during sGVS are shown. These nausea ratings were also significantly different between sGVS and dlPFC stimulation (*P* = 0.0099) as well as between sGVS and sGVS + dlPFC stimulation (*P* = 0.0226)

**Fig. 3:**
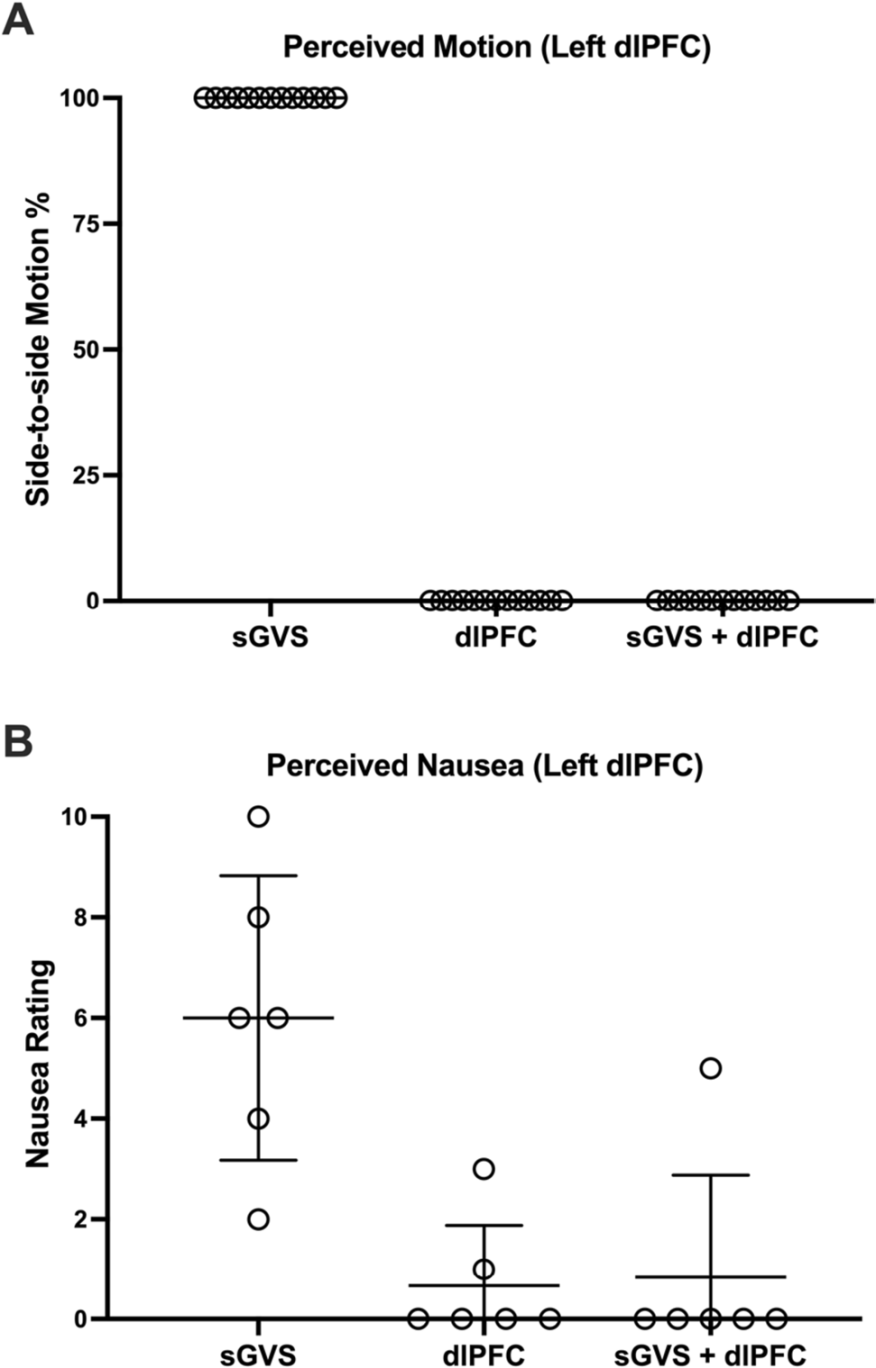
Perceptions of side-to-side motion (**A**) and nausea (**B**) during vestibular stimulation alone (sGVS) (*n* = 13), stimulation of the left dlPFC alone (dlPFC) (*n* = 13) or combined stimulation (sGVS + dlPFC) (*n* = 13). The prevalence of perceived motion was significantly different between sGVS and dlPFC stimulation (*P* = 0.0004) as well as between sGVS and sGVS + dlPFC stimulation (*P* = 0.0004). For the nausea ratings, mean ± SD from the six participants who experienced nausea during sGVS are shown. These nausea ratings were also significantly different between sGVS and dlPFC stimulation (*P* = 0.0281) as well as between sGVS and sGVS + dlPFC stimulation (*P* = 0.0281)

During sGVS, seven of the 20 (35%) right dlPFC participants reported accompanying feelings of mild to severe nausea, ranging from two to seven (mean rating = 4.9 ± 1.9) on the 0–10 scale. Nausea was also seen in six of the 13 (46%) left dlPFC participants, ranging from two to 10 (mean rating = 6.0 ± 2.6). It may be worth noting that none of the five right or the three left dlPFC participants who felt a greater prevalence of leftward sway indicated feelings of nausea. With one exception from each study, any nausea perceptions were completely abolished when sGVS was combined with dlPFC stimulation. One participant rated nausea as 1/10 during combined vestibular and right dlPFC stimulation, compared to 4/10 during sGVS alone, whilst one left dlPFC participant rated nausea as 5/10 during combined stimulation as opposed to 6/10 during sGVS alone. In the case of the latter participant, their experiment had been randomised to include sGVS as the first stimulus and they had indicated a lingering sense of nausea during each of the other stimulation protocols (3/10 for left dlPFC stimulation). A lingering nausea rating of 1/10 was also seen during dlPFC stimulation for another left dlPFC participant. However, by their third stimulation train (the combined stimulation), no nausea was present. Mean data for the perceptions of motion and nausea for each stimulus condition are shown in Figs. 2 and 3. These nausea ratings were significantly different between sGVS and right dlPFC stimulation (*P* = 0.0099), as well as between sGVS and sGVS + right dlPFC combined stimulation (*P* = 0.0226). Similarly, significant differences in nausea ratings across the stimulation protocols were found in the left dlPFC experiments (sGVS vs. left dlPFC stimulation: *P* = 0.0281; sGVS vs. sGVS + left dlPFC combined stimulation: *P* = 0.0281). No other differences were found between those who experienced nausea and those who did not. Another result of interest regards one participant who reported a nausea rating of 10 during the left dlPFC experiment. This participant had been randomised to having combined stimulation first and made no reports of nausea during that stimulation. However, approximately 10 min after commencing sGVS, the participant vomited as a direct result of the nausea.

## Discussion

We have shown, for the first time, that tACS of the dlPFC in awake humans greatly diminishes vestibular illusions of side-to-side movement induced by sGVS. Whilst we hypothesised that dlPFC stimulation may modulate the perception of swaying induced by sGVS, the finding that vestibular illusions were almost completely abolished in all participants was unexpected. Moreover, our finding that dlPFC stimulation further reduced sGVS-evoked nausea in all 13 participants who reported nausea was also unexpected. These results suggest that the cortical processing of vestibular inputs may be blocked by the dlPFC. Precisely where and how this inhibition may occur and the specific neural pathways responsible remain to be elucidated.

The use of tACS itself alters the membrane potential of the cortical neurones in a frequency-dependent manner within the brain region to which it is applied. This thereby alters the probability of action potential generation in phase with the stimulus without changing the overall rate of firing (Antal and Paulus 2013; Elyamany et al. 2021). As such, in stimulating the vestibular apparatus concurrently and by the same means as the dlPFC, the entrained (and ‘heightened’ with peaks and troughs of the sinusoidal current) vestibular activity would appear to be compensated for, and subsequently downregulated again, by the entrained and ‘heightened’ dlPFC activity. This follows a similar principle as seen in the work done by Arshad et al. (2015). The authors used off-vertical axis rotation (OVAR) to induce motion sickness in their participants. After a recovery period, transcranial direct current stimulation was applied to the left posterior parietal cortex via EEG site P3 before and during another OVAR session. This resulted (during cathodal stimulation specifically) in a significant increase in the time taken for participants to develop moderate nausea and a decrease in the time taken for recovery. Results such as these would seem to follow a pattern much the same as the results from our own study—electrical stimulation of a cortical region reducing the motion sickness response. Granted, Arshad et al. (2015) stimulated a region decidedly closer to areas implicated in vestibular processing (i.e. the temporoparietal junction). This may mean that the dlPFC is exerting influence through the same pathway, with a shared key area in which the information is blocked. Possible areas in which area this may be taking place are discussed below.

What is known is that the dlPFC exerts ‘top-down’ control of many functions. Several studies have explored the association of the dlPFC with the vestibular system through vertigo disorders. Patients presenting with persistent postural–perceptual dizziness (PPPD), for example, have a reported decrease in grey matter volume within the dlPFC (Wurthmann et al. 2017), alongside other integrative cortices, each being implicated in multisensory vestibular processing (Becker-Bense et al. 2020; Critchley et al. 2004; zu Eulenburg et al. 2010). This supports the idea that dlPFC function could be associated with persistent vestibular-mediated perceptions of sway and that dlPFC stimulation can influence these perceptions. In such a state, decreased dlPFC volume can be reasonably assumed to lead to decreased activity. Therefore, should dlPFC grey matter volume or, indeed, activity be upregulated in patients presenting with PPPD (thereby returning it to a ‘normal’ state), it can be fairly concluded from our results that a reduction may be found in the severity of the symptoms. Impaired dlPFC activity here would seem to equate to an overactive vestibular perceptual response; therefore, heightened dlPFC activity may very well equate to a diminished vestibular response, as the results presented in our study suggest.

Exactly how the dlPFC influences the perceptions of sway, however, remains unknown. One likely possibility is via direct connexions with vestibular-related areas such as the insular cortex. As previously mentioned, the posterior insula and the adjacent structures play a core role in the vestibular information-processing pathway (Akbarian et al. 1993; Chen et al. 2010; Guldin and Grüsser 1998). The dlPFC has robust interconnections with the insula cortex, albeit most commonly in emotion regulation and conscientiousness (Fu et al. 2021; Gao et al. 2021; Steward et al. 2016). These connexions are primarily found between the dlPFC and the anterior region of the insula, which is not deemed to be part of the PIVC. This is not to say, however, that the dlPFC does not have connexions to the posterior insula too. Indeed, Harricharan et al. (2020) demonstrated a link between these brain regions in emotional appraisal regarding sensory information. With this being said, dlPFC connexions to the posterior insula are far more sparsely noted than connexions to the anterior insula. However, several brain imaging studies with vestibular stimulation have demonstrated anterior insula activation too (Becker-Bense et al. 2020; Bense et al. 2001; Eickhoff et al. 2006; Stephan et al. 2005). Given this evidence as well as its interoceptive properties, the anterior insula may prove influential in vestibular signalling (Lopez and Blanke 2011). Whilst synergistic relationships between the dlPFC and anterior insula have been noted (Critchley et al. 2004; Fu et al. 2021; Ihara et al. 2019; Yuan et al. 2020), interestingly, these structures are known to have an antagonistic relationship as well (Aupperle et al. 2012; Bi et al. 2017; Gao et al 2021). That is, a negative correlation found through imaging analysis in various disease states. Somewhat in line with this, Huang et al. (2021) proposed the idea that the anterior insular cortex was acting as a ‘gate’ for conscious access to sensory information—given its prime location amongst the functional gradients of the brain (Huntenburg et al. 2018)—before being delivered to the dlPFC. Should this hypothesis prove correct, it may have significance in our findings. Granted, in stimulating the dlPFC directly, we approach the proposed insula ‘gate’ in the opposite direction, but the possibility that information can be blocked at this site is still feasible. If upregulating dlPFC activity (through tACS) leads to downregulation of insula activity (thereby ‘gating’ signal propagation—including that from the vestibular apparatus), the dlPFC would be capable of inhibiting vestibular perceptions during sGVS.

Whilst a direct link between the dlPFC and insula is a possible route via which the dlPFC could influence vestibular perceptions, it is possible that other pathways are in play. For example, individuals with a poor psychopathological disposition, such as those with heightened levels of neuroticism and introversion, are more susceptible to developing psychosomatic disorders related to dizziness (Indovina et al. 2014; Tschan et al. 2011). Given the proposed role of the amygdala, which has a key deterministic function in an individual’s level of neuroticism and introversion (Pang et al. 2016), this subcortical structure may also be involved in regulating vestibular-related perceptions. In a similar vein, the amygdala may contribute to hyper-gravity-induced motion sickness, with lesions to this area attenuating the response (Uno et al. 2000). Granted, sGVS-induced nausea does not equate to motion sickness per se, as strictly speaking, motion sickness itself is derived from a visual–vestibular mismatch (Reason 1978). Nevertheless, this connexion further establishes the amygdala’s potential contributions to the vestibular system. Importantly, the amygdala is also found to work synergistically with the dlPFC (Berboth and Morawetz 2021; Siegle et al. 2007), and amygdala–prefrontal connectivity is most commonly noted during emotion regulation. Due to the potential role of the amygdala in motion sickness (a field that, as of yet, has not been extensively studied), this connectivity may also be implicated in the combined stimulation protocol we delivered. It is possible that dlPFC actions on sGVS-induced vestibular perceptions—including nausea—are working via direct connexions between the dlPFC and amygdala.

## Limitations

Whilst our data set included both males (right dlPFC: *n* = 15; left dlPFC: *n* = 7) and females (right dlPFC: *n* = 8; left dlPFC *n* = 6), the number of people who experienced nausea during vestibular stimulation was low. Of the seven participants who reported nausea during the right dlPFC experiments, six were male. It seems somewhat unlikely that this sex disparity would play a role in the obtained results, as it is known that the severity of motion sickness does not vary between sexes (Park and Hu 1999), and such a disparity was not seen during the left dlPFC experiments (of the six who indicated nausea, there was an even distribution of three males and three females), but it may be worth noting. Interestingly, the occurrence and the amount of nausea from our experiments did not correlate with the participants’ perceived sway. This may be attributed to each individual’s personal susceptibility to motion sickness, as a vast array of factors are known to contribute (Golding 2006), though how this may play a part in sGVS-induced nausea remains to be seen.

Further to this, the data are restricted to the age distribution of the participants. Having acquired data from a sample of subjects ranging in age from 21 to 32 years and 21 to 39 years (with a skew towards the younger end of that range; mean: 23.3 ± 2.8 years and mean: 26.4 ± 4.3 years, respectively), our results are exclusive of a large portion of the population. With this being said, susceptibility to motion sickness is known to peak in childhood with a gradual decline through adolescence and adulthood (Huppert et al. 2019; Paillard et al. 2013). For this reason, it could be argued that a young sample may be the most applicable for this study and that age likely does not have a major impact. However, the extent to which age may play a role in our results remains unknown.

Additionally, stimulating the vestibular apparatus through sGVS allows specific targeting of vestibular afferents which other methods of vestibular stimulation cannot provide (Cui et al. 2001; Kaufmann et al. 2002). With this being said, it may be argued that delivering the sinusoidal current to the dlPFC during OVAR or translational movements of the body would have more relevant clinical use. These methods of stimulation more closely resemble the real-world scenarios in which nausea could take place. As such, further exploration using these methods may prove instructive.

Similar to the specificity of sGVS, tACS applied over the dlPFC will specifically stimulate that area of the brain. However, having placed the reference electrode on the nasion, stimulation travelled across the ventromedial prefrontal cortex (vmPFC), which may have had an impact on its resting membrane potential. In humans, the vmPFC is known to exert control on cardiovascular activity during resting conditions (Wong et al. 2007), which are dissimilar to the electrical stimulation conditions employed in this project. As such, the notion that the vmPFC may have influenced the perceptual results during combined sGVS and tACS remains doubtful. Regardless, the vmPFC is worth noting, especially with regard to vestibular, dlPFC (Sesa-Ashton et al. 2022; Wong et al. 2023) and a possible combined influence on sympathetic activity—an exciting avenue for further research.

## Conclusions

In this novel study, we established a link between the vestibular system and the dlPFC in the control of vestibular illusions. Perceptions of sway induced by sinusoidal stimulation of the vestibular apparatus were almost completely abolished during a combined dlPFC and vestibular stimulation protocol. Moreover, nausea was also essentially abolished in those who reported it during sGVS. The precise mechanisms of this interaction remain unknown, though we deem it likely that the dlPFC is exerting top-down inhibitory control on the vestibular information-processing pathway.

## Data Availability

The data are available on reasonable request.
